# Gluconeogenesis combats cancer: opening new doors in cancer biology

**DOI:** 10.1038/cddis.2015.245

**Published:** 2015-09-03

**Authors:** M W Khan, P Chakrabarti

**Affiliations:** 1 Division of Cell Biology and Physiology, CSIR-Indian Institute of Chemical Biology, Kolkata, West Bengal, India

Research from our group has demonstrated a novel mechanism of cancer cell death by mTOR (mechanistic target of rapamycin) inhibitors, often used in hepatocellular (HCC) and renal cell carcinomas (RCC) therapy, by augmenting the gluconeogenesis pathway. Our results further suggest that upregulation of the gluconeogenic pathway creates a metabolic stress where bulk of the glucose is exported outside the cells creating a 'futile' cycle for glucose that in turn detrimentally affects the cell survival. The work is in-press and is scheduled to be published in the new NPG journal *Cell Death Discovery*.^1^

Metabolic reprogramming, now considered a hallmark of cancer, has become a major research area in cancer biology in the past decade.^2, 3^ An expanding compendium of metabolic pathways associated with oncogenesis, tumor progression and metastasis have been and being discovered. Otto Warburg's seminal observation of proliferating ascites tumor cells converting the bulk of glucose carbon to lactate, even in normoxia, was among the early experimental evidence of rewiring of glucose metabolism in cancer cells.^4^ Thus bypassing the mitochondrial respiration in oxygen-rich conditions albeit enhanced rate of glycolysis, now termed aerobic glycolysis, is one of the key features of metabolic reprogramming intrinsic to cancer cells. Indeed, alteration in the multiple metabolic shuts including pentose phosphate pathway, serine-glycine pathway and several control points in the glycolytic pathway such as hexokinase 2, pyruvate kinase M2 and lactate dehydrogenase stimulate glycolysis and provide prosurvival cues. Blockade of pyruvate flux into mitochondria via mitochondrial pyruvate carrier in parallel further accentuates the metabolic rewiring. ‘Glucose addiction' thus endows cancer cells with enhanced metabolic flexibility for their anabolic requirements to support growth, proliferation and survival.^5, 6^

Although an in-depth understanding of glycolytic switch in cancer metabolism has grown in recent years, however gluconeogenesis, which drives the metabolic flux in parallel and opposite to glycolysis, has got little attention.^7^ Gluconeogenesis is defined metabolically as the production of glucose from non-carbohydrate carbon sources such as pyruvate, lactate, glycerol and gluconeogenic amino acids. Thus, it is imperative that concomitant activation or downregulation of glycolysis and gluconeogenesis would cause a metabolic stress, which might in turn disrupt the metabolic rewiring of cancer cells. Targeting glycolysis/gluconeogenesis pathways as a consequence of metabolic reprogramming therefore is an attractive therapeutic strategy because it is central to the growth and survival of cancer cells.^8^

The objective of our work was to elucidate the role of mTOR-mediated metabolic reprogramming and its consequences in proliferation and survival of human cancer cells.The mTOR kinase is the catalytic subunit of two functionally distinct complexes, mTORC1 and mTORC2, which serves as a central regulator for promoting cell growth, proliferation and survival.^9, 10^ Hyper-activation of mTOR signaling pathway is often associated with cancer cell growth and survival including HCC and RCCand the use of mTORC1 inhibitors rapamycin and its analogshas been approved for the treatment of advanced RCC, albeit with modest success.On the other hand, ATP competitive mTOR inhibitors that fully inhibit both the mTOR complexes exhibit stronger antitumor effects suggesting a potential contribution of mTORC2 in tumor progression.^11, 12^

In this context, we took both pharmacological as well as RNAi approaches to block specifically mTORC1, mTORC2 or both followed by biochemical analysis of metabolic ramifications of inhibition mTOR in cancer cells, specifically HCC and RCC. Initial experiments suggested a potential sterol regulatory element-binding protein-independent downregulation of lipogenesis, a well established mTOR-dependent metabolic pathway. Wenext focused on transcriptional activation of forkhead box O following mTOR inhibition as a potential mediator of metabolic changes. By upregulating *G6PC* and *PCK1*, the two rate-limiting enzymes of gluconeogenesis, forkhead box O serves as a strong inducer of glucose production. Surprisingly, instead of glucose consumption being reduced, blocking mTOR led to an increase in glucose consumption and lactate production. Subsequent examination of mitochondrial metabolites showed that the increased glucose consumption was not leading to increased mitochondrial oxidative phosphorylation, but instead these metabolites were being shunted off into gluconeogenesis. Thus, mTOR inhibition arguably creates a 'futile' cycle where glucose consumption is increased, only to have it be broken down and then recycled through gluconeogenesis (Figure 1). The detrimental effect of this cycle was shown to be significant, as reduction in *PCK1* but not *G6PC* after mTOR inhibition could prevent gluconeogenesis from increasing, preventing this futile cycle from initiating, and substantially reduce apoptosis after mTOR inhibition. Finally, transcriptomic analysis was conducted for metabolic genes involved in gluconeogenesis, across multiple tumor types, and HCC and RCC was found to have reduced expression of genes for multiple enzymes found in this pathway. Interestingly, mTOR inhibition could only enhance cell death in HCC and RCC cell lines whereas in cell lines where mTOR inhibition could not lead to the activation of gluconeogenesis did not undergo significant cell death. Our findings thus uncover an important role of mTOR in cancer biology and possibly explain the phenomenon as to why mTOR inhibitors are effective in certain cancer types only.

## Figures and Tables

**Figure 1:**
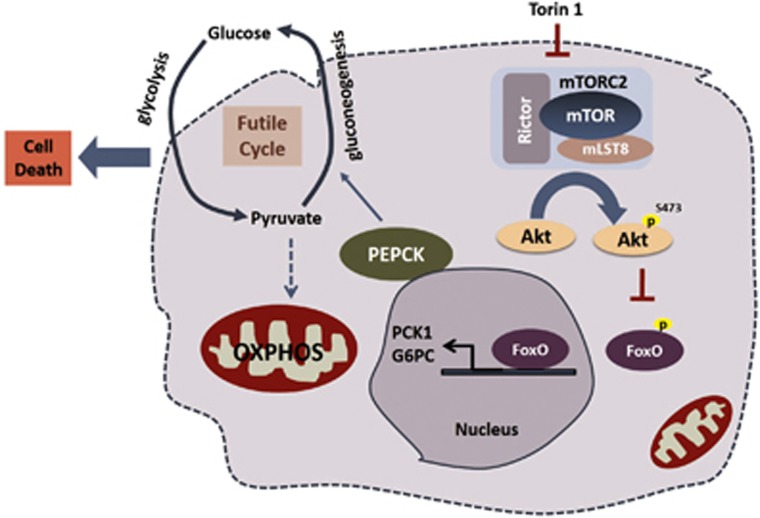
Torin 1-mediated mTOR leads to cancer cell death via upregulation of gluconeogenesis: Torin 1 treatment in inhibition of mTOR complex 2 that results in degradation of protein kinase B (PKB/Akt). Loss of Akt leads to enhanced retention of forkhead box O (FoxO) protein in the nucleus where it transcribes its downstream effectors PCK1 and G6Pase thereby leading to increased protein levels of PEPCK. Enhanced PEPCK levels results in increased glucose output by the cell. As mTOR inhibition blockades the entry of glucose derived pyruvate into the mitochondria and enhanced PEPCK leads to increased glucose production, this leads to a ‘futile cycle' of glucose carbons in and out of the cell without being utilized by the cell further leading to metabolic stress and cell death
